# High BANCR expression is associated with worse prognosis in human malignant carcinomas: an updated systematic review and meta-analysis

**DOI:** 10.1186/s12885-020-07177-6

**Published:** 2020-09-09

**Authors:** Shixu Fang, Zhou Liu, Qiang Guo, Cheng Chen, Xixian Ke, Gang Xu

**Affiliations:** Department of Thoracic Surgery, The Affiliated Hospital of Zunyi Medical University, 149 Dalian Road, Zunyi, 563000 Guizhou China

**Keywords:** Long noncoding RNA, BANCR, Cancer, Prognosis, Meta-analysis

## Abstract

**Background:**

BRAF-activated noncoding RNA (BANCR) is aberrantly expressed in various tumor tissues and has been confirmed to function as a tumor suppressor or oncogene in many types of cancers. Considering the conflicting results and insufficient sampling, a meta-analysis was performed to explore the prognostic value of BANCR in various carcinomas.

**Methods:**

A comprehensive literature search of PubMed, Web of Science, EMBASE, Cochrane Library and the China National Knowledge Infrastructure (CNKI) was conducted to collect relevant articles.

**Results:**

The pooled results showed a strong relationship between high BANCR expression and poor overall survival (OS) (HR (hazard ratio) =1.60, 95% confidence interval (CI): 1.19–2.15, *P* = 0.002) and recurrence-free survival (RFS) (HR = 1.53, 95% CI: 1.27–1.85, *P* < 0.00001). In addition, high BANCR expression predicted advanced tumor stage (OR (odds ratio) =2.39, 95% CI: 1.26–4.53, *P* = 0.008), presence of lymph node metastasis (OR = 2.03, 95% CI: 1.08–3.83, *P* = 0.03), positive distant metastasis (OR = 3.08, 95% CI: 1.92–4.96, *P* < 0.00001) and larger tumor sizes (OR = 1.63, 95% CI: 1.09–2.46, *P* = 0.02). However, no associations were found for smoking status (OR = 1.01, 95% CI: 0.65–1.56, *P* = 0.98), age (OR = 0.88, 95% CI: 0.71–1.09, *P* = 0.236) and sex (OR = 0.91, 95% CI: 0.72–1.16, *P* = 0.469). The sensitivity analysis of OS showed that the results of each publication were almost consistent with the combined results, and the merged results have high robustness and reliability.

**Conclusions:**

The results showed that elevated BANCR expression was associated with unfavorable prognosis for most cancer patients, and BANCR could serve as a promising therapeutic target and independent prognostic predictor in most of cancer types.

## Background

Currently, cancer remains one of the major public health concerns worldwide [1]. Approximately 1,762,450 new cancer cases and 606,880 cancer deaths were predicted to occur in the United States in 2019 [2]. Notably, due to the rapid advancement of cancer research, treatment and diagnostic methods, cancer mortality has continuously decreased by a total of 27% in the last two decades [3]. In spite of this, the 5-year relative survival rate of patients is still unsatisfactory [4]. When cancer is diagnosed, many patients are already in the middle and late stages of the disease, and there is still no ideal effective treatment. Therefore, it is critical to explore specific and sensitive therapeutic targets and promising prognostic biomarkers for the effective treatment of cancer.

Increasing studies have suggested that long noncoding RNAs (lncRNAs), which are transcripts longer than 200 nucleotides that do not have the ability to code proteins, play vital roles in multifarious biological processes, including cell differentiation, growth, apoptosis, cell cycle and metabolism [5]. Moreover, abnormal lncRNA expression has been observed in various tumor tissues and is involved in the proliferation, invasion and metastasis of tumor cells [6–8]. A growing number of publications have revealed the great application value of long noncoding RNAs, including MALAT1 [9], CRNDE [10], ZEB1-AS1 [11], etc., in targeted treatment and cancer prognosis.

By using RNA-sequencing, Flockhart et al. originally found that BRAF-activated noncoding RNA (BANCR), a 693-bp lncRNA located on chromosome 9, was overexpressed in melanoma cells. Additionally, accumulating studies have suggested that BANCR is correlated with the metastasis and invasion of multiple tumor cells and could function as a prognostic biomarker for cancers such as gastric cancer [12, 13], hepatocellular carcinoma [14–17], renal cell carcinoma and non-small cell lung cancer [18, 19]. However, due to the small sample size and discrepant conclusions among those studies, the association of BANCR expression with the prognosis of patients is still undefined. Thus, a meta-analysis was performed to investigate the prognostic value of BANCR in various cancers.

## Methods

### Literature search strategies

A literature search was conducted in the electronic databases of PubMed, Cochrane Library, EMBASE, Web of Science and the Chinese National Knowledge Infrastructure (CNKI) by using the following terms: (“BANCR” OR “Lnc RNA BANCR” OR “lncBANCR” OR “BRAF-activated non-coding RNA”) AND (“neoplasm” OR “carcinoma” OR “tumor” OR “cancer”). The latest literature search was performed up to July 25, 2019.

### Inclusion and exclusion criteria

The selection of studies was completed independently by two researchers. The inclusion criteria were as follows: (a) studies investigated the correlation of BANCR expression with the survival outcomes and clinical prognosis of cancer patients; (b) patients were classified into a high expression group and a low expression group in accordance with the primary literature; (c) the expression level of BANCR was detected by validated techniques; (d) publications provided sufficient and usable data to calculate the OR and HR; and (e) studies published in English or Chinese. The exclusion criteria were as follows: (a) publications exploring the molecular biological mechanisms of BANCR but not investigating the relationship between the expression level of BANCR and the prognosis of cancer patients; (b) reviews and meta-analyses, letters, animal studies, and conference literature; (c) studies without enough data to perform prognostic analysis; and (d) duplicate publications.

### Data extraction and quality assessment

The data were independently extracted by two investigators (FSX and LZ), including first author’s name, publication date, cancer type, sample size, overall survival (OS), recurrence-free survival (RFS), disease-free survival (DFS), TNM stage, tumor size, distant metastasis (DM), histological grade, lymph node metastasis (LNM), depth of invasion, smoking status, follow-up time of patients, detection methods of BANCR and HR, age and sex. The Newcastle-Ottawa Scale (NOS) was used to assess the quality of the included articles, and high-quality studies had NOS scores greater than 6 [20].

### Statistical analysis

The meta-analysis was conducted to calculate the pooled ORs and HRs with corresponding 95% CIs by using Review Manager 5.3 software (Cochrane Collaboration, London, UK) and STATA 12.0 software (Stata Corp., College Station, TX). A random-effects model was adopted when *I*^*2*^>50%, which indicated significant heterogeneity among the enrolled studies, otherwise, a fixed-effects model was applied. Publication bias was assessed by using funnel plots and Begg’s test. When significant heterogeneity existed, subgroup analysis was conducted to explore the source of heterogeneity. Sensitivity analysis was carried out to test the reliability and stability of the results by excluding each of the included studies one by one and then combining the effect sizes to determine whether the result of a single study significantly affected the overall result. Especially, when survival data could not be directly extracted and only Kaplan-Meier curves were provided in the primary articles, the Engauge Digitizer tool (Version 4.1) was used to extract the time-dependent survival rate from the Kaplan-Meier curves, and the HRs and 95% CIs were calculated according to the method in [21]. Statistical significance was considered when *P*<0.05.

## Results

### Study characteristics

**Fig. 1:**
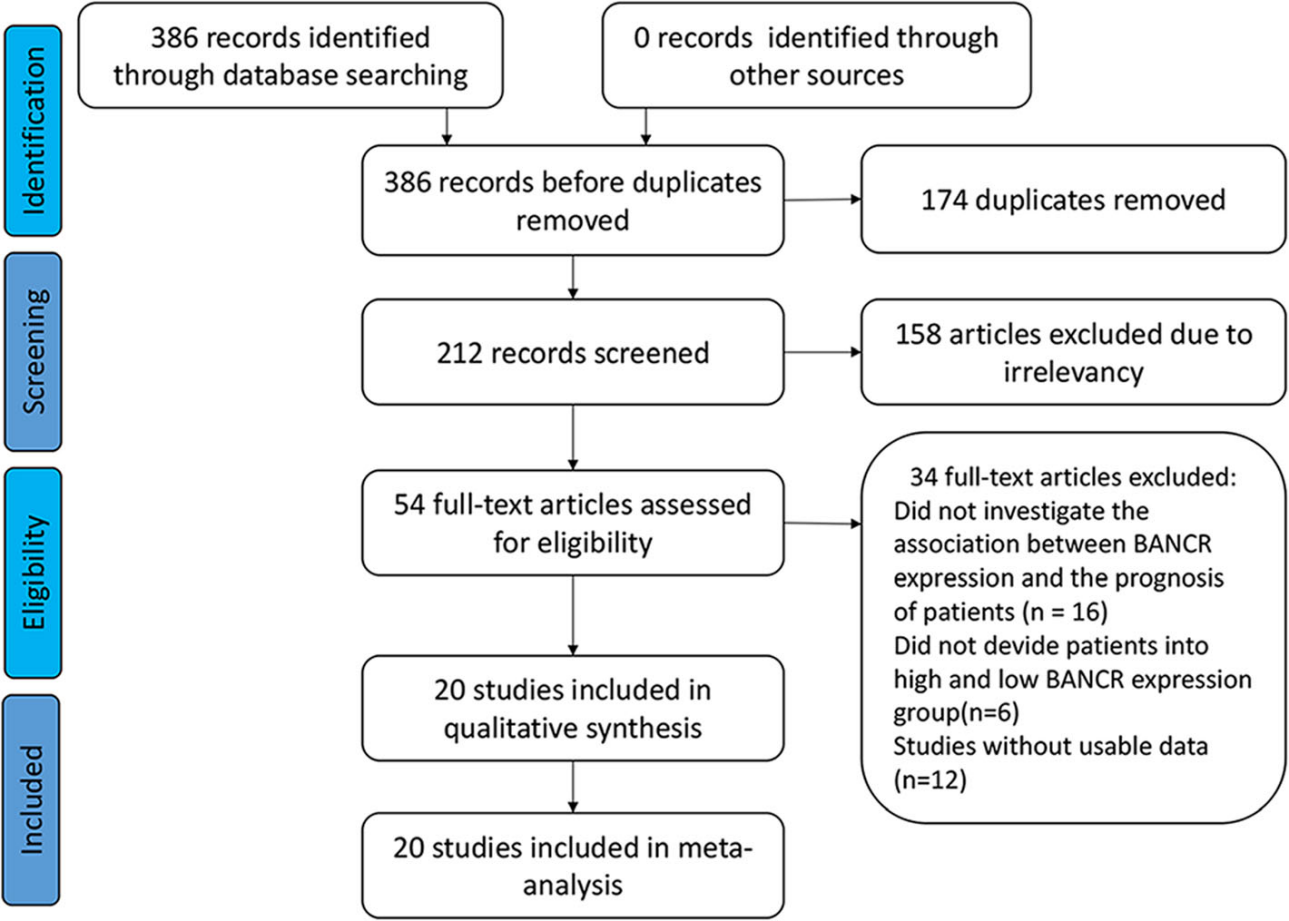
Flow diagram of the study search and selection in this meta-analysis

A total of 386 studies were identified from the databases; among them, 174 duplicate studies were excluded, and 158 studies were omitted after reading the abstracts and full texts. Furthermore, 16 publications did not investigate the association between BANCR expression and the prognosis of patients, 6 publications did not divide patients into high and low BANCR expression groups, and 12 publications lacked usable data. Finally, 20 eligible studies were included for qualitative and quantitative synthesis (Fig. 1).

Of these 20 studies with 1997 patients, 19 studies with 1847 patients were from China, and 1 study comprising 150 patients was from Iran [22]. The publication years ranged from 2014 to 2019, and the expression levels of BANCR were all detected by qRT-PCR for the following cancer types: lung cancer [19], hepatocellular carcinoma [15–17], osteosarcoma [23], papillary thyroid cancer [24–27], gastrointestinal cancer [28, 29], bladder cancer [30], malignant melanoma [31], breast cancer [32, 33], clear cell renal cell carcinoma [18], esophageal squamous cell carcinoma and endometrial cancer (details in Table 1) [22, 35, 36]. The NOS scores are presented in Table 2.

**Table 1:** Basic features of the publications included in this meta-analysis (*n* = 20)

			cancer type	No. of patients	BANCR expression								detection method	survival analysis		HR statistics	hazard ratios (95% CI)	follow-up (month)
Study (Reference)	year	country			high				low						cut-off			
					total	LNM	HTS	DM	total	LNM	HTS	DM						
Guo Q [34]	2014	China	CRC	60	18	14	16	–	42	13	16	–	qRT-PCR	OS	mean-value	NA	NA	NA
He A [30]	2016	China	Bladder cancer	54	19	1	9	–	35	3	30	–	qRT-PCR	OS	NA	NA	NA	NA
Liu T [27]	2018	China	PTC	30	17	4	–	–	13	6	–	–	qRT-PCR	OS	mean-value	directly	NA	60
Li L [12]	2015	China	GC	184	92	60	67	12	92	43	51	0	qRT-PCR	OS	median	directly	1.511(1.025–2.227)	100
Liao T [24]	2017	China	PTC	92	29	14	4	–	63	30	22	–	qRT-PCR	–	NA	NA	NA	NA
Liu Z [35]	2016	China	ESCC	142	71	57	41	30	71	33	23	19	qRT-PCR	OS	median	directly	2.24(1.05–4.76)	60
														DFS	median	directly	3.45(1.92–6.20)	
Lou K [33]	2018	China	BC	65	34	24	18	–	31	13	6	–	qRT-PCR	OS	NA	SC	1.39(0.86–2.25)	120
														DFS			1.48(0.83–2.64)	
Shen X [29]	2017	China	CRC	116	53	32	–	–	53	17	–	–	qRT-PCR	OS	median	directly	2.24(1.22–4.11)	70
Sun M [19]	2014	China	NSCLC	113	53	19	11	–	60	40	30	–	qRT-PCR	OS	fold change	SC	0.5(0.26–0.94)	40
														DFS			0.34(0.18–0.64)	
Wang D [36]	2016	China	EC	30	15	6	7	–	15	1	1	–	qRT-PCR	OS	median	NA	NA	NA
Wang H [37]	2016	China	HCC	108	43	29	35	–	65	23	20	–	qRT-PCR	OS	mean-value	NA	NA	NA
Jiang J [32]	2018	China	BC	216	125	63	60		91	17	18		qRT-PCR	OS	median	NA	1.585(1.298–1.935)	60
														RFS			1.532(1.272–1.844)	
Zhang J [26]	2018	China	PTC	60	17	6	–	–	43	30	–	–	qRT-PCR	OS	NA	NA	NA	NA
Sadeghpour [22]	2018	Iran	ESCC	150	75	–	38	41	75	–	17	10	qRT-PCR	OS	NA	NA	NA	NA
Peng Z [23]	2015	China	Osteosarcoma	84	42	–	30	20	42	–	16	14	qRT-PCR	OS	median	directly	2.934(1.12–7.67)	60
Zhao N [16]	2018	China	HCC	46	23	–	7	–	23	–	4	–	qRT-PCR	OS	mean-value	NA	NA	NA
Zhou T [17]	2016	China	HCC	109	54	–	37	–	55	–	21	–	qRT-PCR	OS	median	SC	4.24(1.32–13.61)	60
Su S [38]	2015	China	retinoblastoma	60	30	–	–	–	30	–	–	–	qRT-PCR	OS	median	directly	2.9(1.05–8.03)	60
Xue S [18]	2018	China	ccRCC	62	–	–	–	–	–	–	–	–	qRT-PCR	OS	NA	SC	0.77(0.24–2.47)	60
Chen Q [39]	2018	China	ESCC	80	39	30	–	–	41	16	–	–	qRT-PCR	NA	median	NA	–	NA

**Note.** BRAF-activated noncoding RNA; *No*. Number; Total: total patients in high expression group or low expression group; *NSCLC* Non-small cell lung cancer; *HCC* Hepatocellular carcinoma; *CRC* Colorectal cancer; *BL* Bladder cancer; *BC* Breast cancer; *ccRCC* Clear cell renal cell carcinoma; *GC* Gastric cancer; *LNM* Lymphatic node metastasis; *DM* Distant metastasis; *HTS* High tumor stage (III,IV); *NA* Not available; *qRT-PCR* Quantitative reverse transcription-polymerase chain reaction; *ESCC* Esophagus cancer; *EC* Endometrial Cancer; *SC* Survival curve; directly: HR was extracted dirrectly from article; *PTC* Thyroid Carcinoma; *OS* Overall survival; *DFS* Disease free survival; *RFS* Recurrence free survival

**Table 2:** Quality assessment of eligible studies (Newcastle-Ottawa Scale) (NOS score)

Author (Reference)	Country	Selection				Comparability	Outcome			Total
		Adequate of case definition	Representativeness of the cases	Selection of Controls	Definition of Controls	Comparability of cases and controls	Ascertainment of exposure	Samemethod of ascertainment	Non-Responserate	
Guo Q [34]	China	*	*	*	*	**	*	*	*	9
He A [30]	China	*	*	*	*	*	*	*	*	8
Liu T [27]	China	*	*	*	*	**	*	*	*	6
Li L [12]	China	*	*	*	*	**	*	*	*	9
Liao T [24]	China	*	*	*	*	**	*	*	*	9
Liu Z [35]	China	*	*	*	*	**	*	*	*	9
Lou K [33]	China	*	*	*	*	**	*	*	*	9
Shen X [29]	China	*	*	*	*	*	*	*	NA	7
Sun M [19]	China	*	*	*	*	**	*	*	*	9
Wang D [36]	China	*	*	*	*	**	*	*	*	9
Wang H [37]	China	*	*	*	*	**	*	*	*	9
Jiang J [32]	China	*	*	*	*	**	*	*	*	9
Zhang J [26]	China	*	*	*	*	**	*	*	*	9
Sadeghpour [22]	Iran	*	*	*	*	*	*	*	*	8
Peng Z [23]	China	*	*	*	*	**	*	*	*	9
Zhao N [16]	China	*	*	*	*	**	*	*	*	9
Zhou T [17]	China	*	*	*	*	**	*	*	*	9
Su S [38]	China	*	*	*	*	**	*	*	*	9
Xue S [18]	China	*	*	*	*	**	*	*	*	9
Chen Q [39]	China	*	*	*	*	*	*	*	*	8

Note: *NA* Not available

### The association of BANCR with OS

**Fig. 2:**
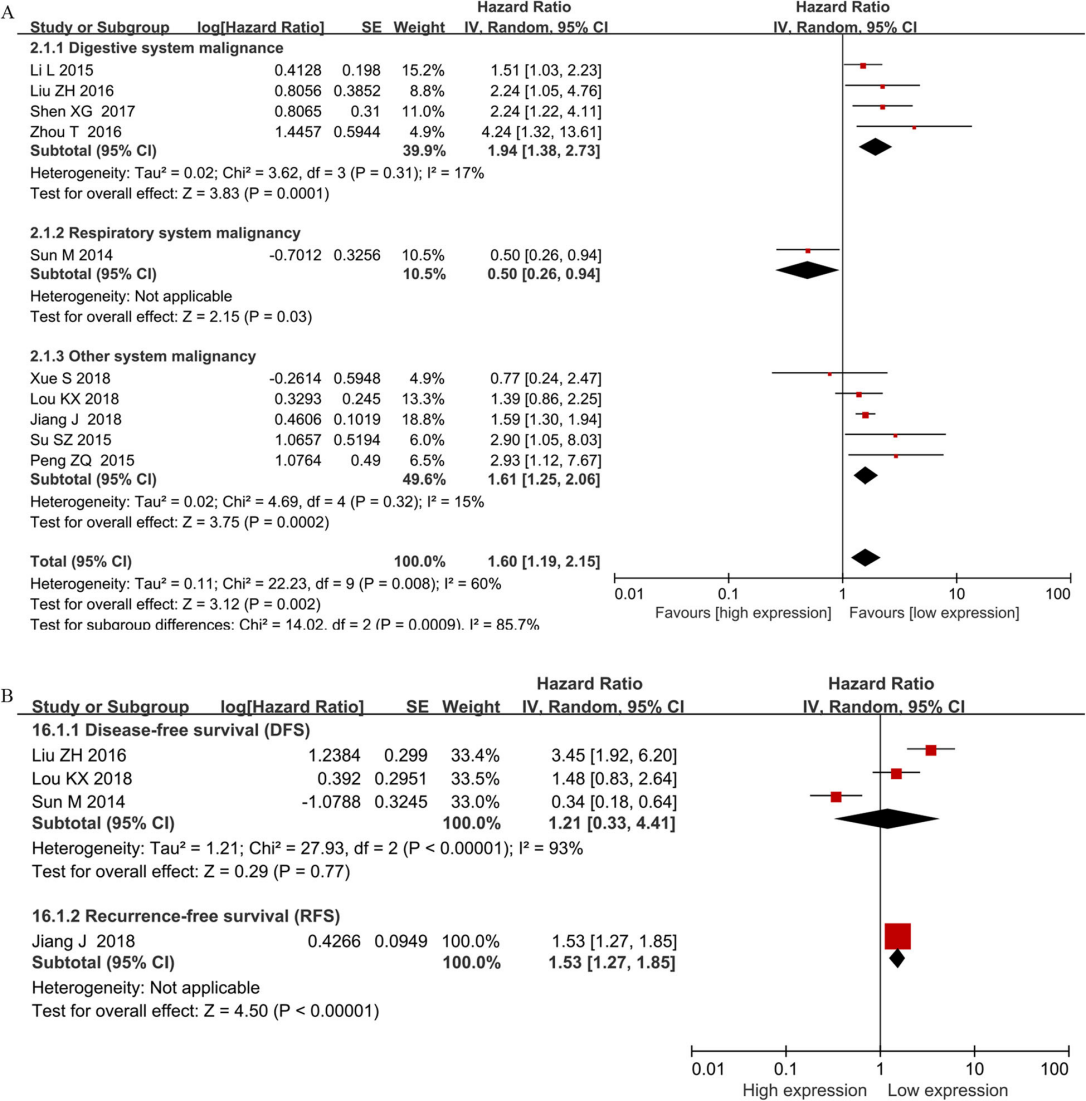
Forest plot showing the relationship between BANCR expression and OS, DFS and RFS in cancers. Note: overall survival (OS); disease-free survival (DFS); recurrence-free survival (RFS); BANCR: BRAF-activated noncoding RNA; CI: confidence interval; Random: random-effects model; The random-effects model was adopted. The square size of individual studies represented the weight of the study. Vertical lines represent 95% CI of the pooled estimate. The diamond represents the overall summary estimate, with the 95% CI given by its width

A total of 10 studies comprising 1151 patients were included in the analysis of the relationship between BANCR and OS. The random-effects model was applied due to marked heterogeneity (*I*^*2*^ = 60%, *P* = 0.008). The pooled results supported the conclusion that patients with high BANCR expression tended to have shorter overall survival (HR = 1.60, 95% CI: 1.19–2.15, *P* = 0.002, Fig. 2a). Moreover, subgroup analysis was conducted to explore the sources of heterogeneity based on cancer type, the level of BANCR expression (high BANCR expression vs. low BANCR expression), the method of HR extraction (direct / indirect extraction), sample size (less / more than 100 patients) and NOS score (score of 9 / less than 9). A strong correlation was revealed between high BANCR expression and poor OS for cancers in the digestive system (HR = 1.94, 95% CI, 1.38–2.73; *P* = 0.0001), for HRs extracted directly from articles (HR = 1.69, 95% CI, 1.44–1.99; *P* < 0.00001), for HRs from multivariate analysis (HR = 1.71, 95% CI, 1.47–2.02; *P* < 0.00001), for high BANCR expression group (HR = 1.72, 95% CI, 1.48–1.98; *P* < 0.00001), for studies with less than 100 patients (HR = 1.62, 95% CI, 1.11–2.35; *P* = 0.05) and for studies with more than 100 patients (HR = 1.57, 95% CI, 1.07–2.31; *P* = 0.02). No correlation between BANCR expression and OS was found for non-digestive system cancers (HR = 1.35, 95% CI, 0.86–2.13; *P* = 0.20), for HRs from univariate analysis (HR = 0.84, 95% CI, 0.41–1.75; *P* = 0.65) or HRs extracted indirectly from articles (HR = 1.15, 95% CI, 0.52–2.56; *P* = 0.73). Detailed results are shown in Table 3. The poor prognosis related to BANCR was also identified by the positive association between high BANCR expression and short DFS (HR = 1.21, 95% CI: 0.33–4.41, *P* = 0.77) and RFS (HR = 1.53, 95% CI: 1.27–1.85, *P* < 0.00001) (Fig. 2b**)**.

**Table 3:** Subgroup analysis of BANCR expression and overall survival (OS) in cancer patients

		No. of studies		No. of patients		Pooled HR (95% CI)		Heterogeneity	
						Fixed	Random	*I*^*2*^*(%)*	*P-value*
**Overall survival**		10		1151		1.56 (1.35–1.81)	1.60 (1.19–2.15)	60	0.008
Cancer type									
**Digestive system**		4		551		1.87 (1.40–2.50)	1.94 (1.38–2.73)	17	0.31
GC		1		184		1.51 (1.03–2.23)	1.51 (1.03–2.23)	–	–
ESCC		1		142		2.24 (1.05–4.76)	2.24 (1.05–4.76)	–	–
HCC		1		109		4.24 (1.32–13.61)	4.24 (1.32–13.61)	–	–
CRC		1		116		2.24 (1.22–4.11)	2.24 (1.22–4.11)	–	–
**Non-digestive system**		6		600		1.47 (1.24–1.74)	1.35 (0.86–2.13)	70	0.005
**Respiratory system**		1		113		0.5 (0.26–0.54)	0.5 (0.26–0.54)	–	–
NSCLC		1		113		0.5 (0.26–0.54)	0.5 (0.26–0.54)	–	–
**Other system**		5		487		1.59 (1.34–1.90)	1.61 (1.25–2.06)	15	0.32
BC		2		281		1.55 (1.29–1.87)	1.55 (1.29–1.87)	0	0.62
Osteosarcoma		1		84		2.93 (1.12–7.67)	2.93 (1.12–7.67)	–	–
retinoblastoma		1		60		2.90 (1.05–8.03)	2.90 (1.05–8.03)	–	–
ccRCC		1		62		0.77 (0.24–2.47)	0.77 (0.24–2.47)	–	–
**Analysis method**									
Univariate analysis		3		238		0.95 (0.66–1.37)	0.84 (0.41–1.75)	68	0.04
Multivariate analysis		7		911		1.71 (1.47–2.02)	1.79 (1.47–2.18)	11	0.34
**HR estimation method**									
Indirectly		4		349		1.07 (0.76–1.52)	1.15 (0.52–2.56)	76	0.006
Directly		6		802		1.69 (1.44–1.99)	1.69 (1.44–1.99)	0	0.49
**number of patients**									
more than 100		6		880		1.56 (1.33–1.82)	1.57 (1.07–2.31)	70	0.005
less than 100		4		271		1.62 (1.11–2.35)	1.71 (1.01–2.90)	36	0.2
**BANCR expression level**									
high expression		8		976		1.68 (1.45–1.96)	1.71 (1.44–2.03)	6	0.38
low expression		2		175		0.55 (0.31–0.96)	0.55 (0.31–0.96)	0	0.52
**Quality scores**									
Score = 9		8		973		1.54 (1.32–1.80)	1.61 (1.15–2.24)	64	0.007
Score < 9		2		178		1.78 (1.04–3.06)	1.48 (0.53–4.11)	61	0.008
**DFS**		3		320		1.29 (0.91–1.82)	1.21 (0.33–4.41)	93	0.00001
**RFS**		1		216		1.53 (1.27–1.85)	1.53 (1.27–1.85)	–	–

**Note**: *BANCR* BRAF-activated noncoding RNA; *OS* Overall survival; *DFS*: disease-free survival; *PFS* Progression-free survival; *Random* Random effects; *Fixed* Fixed effects; directly: HR was extracted directly from the primary articles; indirectly: HR was extracted indirectly from the primary articles; *NSCLC* Non-small cell lung cancer; *HCC* Hepatocellular carcinoma; *CRC* Colorectal cancer; *BC* Breast cancer; *ccRCC* Clear cell renal cell carcinoma; *GC* Gastric cancer; *LNM* Lymphatic node metastasis; *DM* Distant metastasis; *HTS* High tumor stage (III,IV);*NA* Not available; *ESCC* Esophagus cancer; directly: HR was extracted directly from article; *OS* Overall survival; *DFS* Disease free survival; RFS: recurrence free survival

### The association of BANCR with TNM stage

**Fig. 3:**
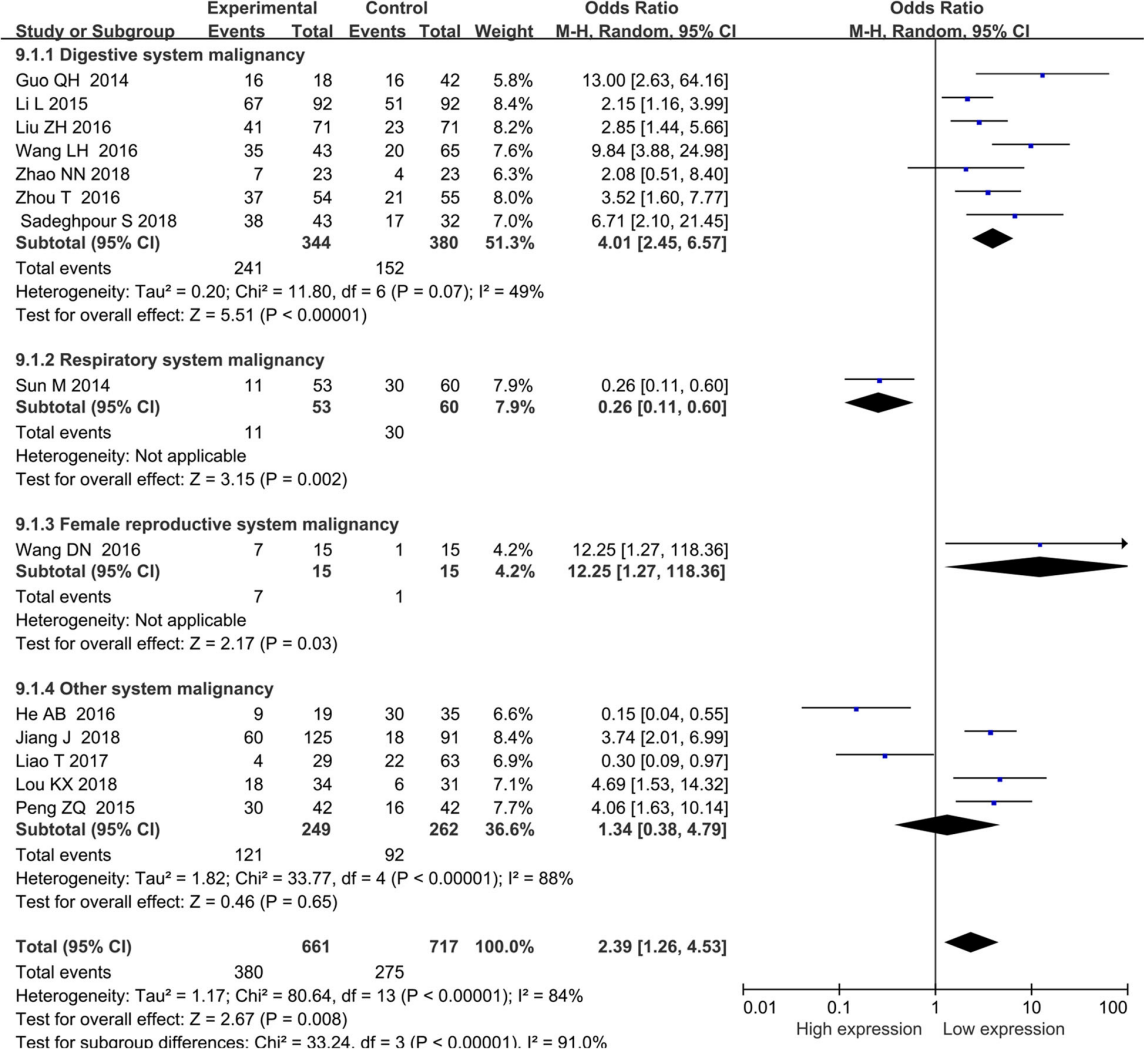
Forest plot of the relationship between BANCR expression and TNM stage. Note: BANCR: BRAF-activated noncoding RNA; CI: confidence interval; Random: random-effects model. The random-effects model was adopted. The square size of individual studies represented the weight of the study. Vertical lines represent 95% CI of the pooled estimate. The diamond represents the overall summary estimate, with the 95% CI given by its width

Fourteen studies including 1378 patients were enrolled to investigate the association of BANCR expression level with TNM stage. The random-effects model was adopted, and subgroup analysis was carried out due to significant heterogeneity (*I*^*2*^ = 83.9%, *P* < 0.00001). The pooled OR showed a strong association between high BANCR expression and advanced tumor stage (HR = 2.39, 95% CI: 1.26–4.53, *P* < 0.001). According to the results of the subgroup analysis, a strong association between high BANCR expression and advanced TNM stage for digestive system cancers (HR = 4.01, 95% CI: 2.45–6.57, *P* < 0.00001) and female reproductive system cancers (HR = 12.25, 95% CI: 1.27–118.37, *P* = 0.03) was found; a negative association for non-small cell lung cancer (HR = 0.26, 95% CI: 0.11–0.61, *P* = 0.002) was found; And no association was found for other system cancers (HR = 1.30, 95% CI: 0.40–4.27, *P* = 0.15) (Fig. 3).

### The association of BANCR with other clinicopathological parameters

**Fig. 4:**
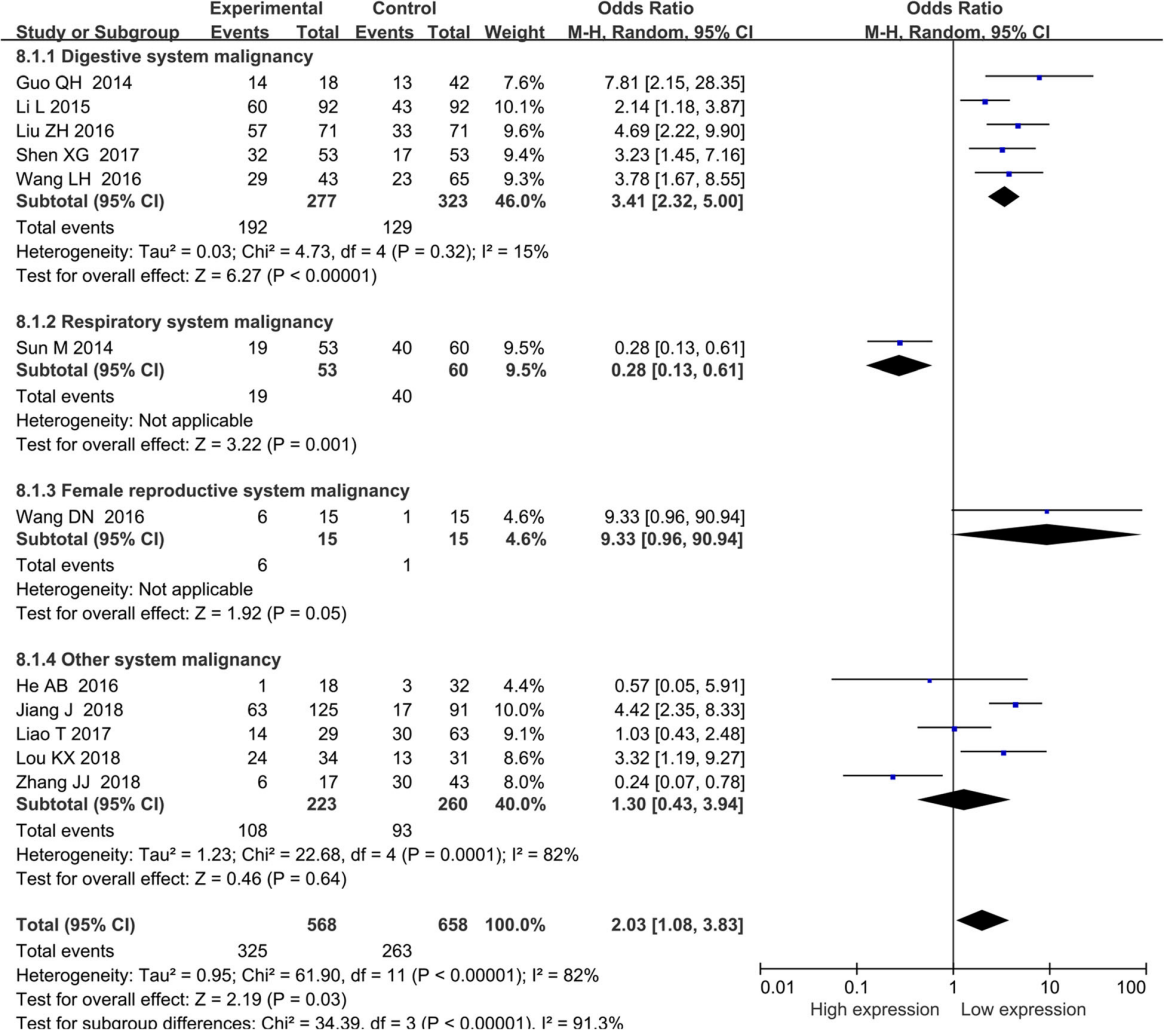
Forest plot of the relationship between BANCR expression and lymph node metastasis (LNM). Note: BANCR: BRAF-activated noncoding RNA; CI: confidence interval; Random: random-effects model. The random-effects model was adopted. The square size of individual studies represented the weight of the study. Vertical lines represent 95% CI of the pooled estimate. The diamond represents the overall summary estimate, with the 95% CI given by its width

**Fig. 5:**
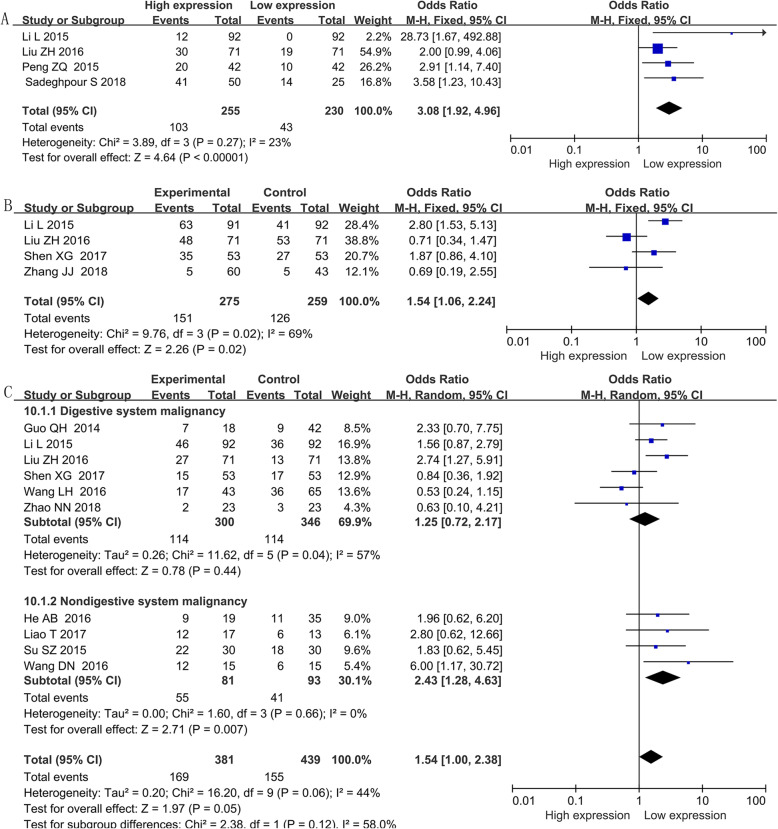
Forest plot of the relationship between BANCR and distant metastasis, invasion depth and histological grade. Note: (**a**): distant metastasis; (**b**): invasion depth; (**c**): histological grade. BANCR: BRAF-activated noncoding RNA; CI: confidence interval; Fixed: fixed-effects model. The fixed-effects model was adopted. The square size of individual studies represented the weight of the study. Vertical lines represent 95% CI of the pooled estimate. The diamond represents the overall summary estimate, with the 95% CI given by its width

**Fig. 6:**
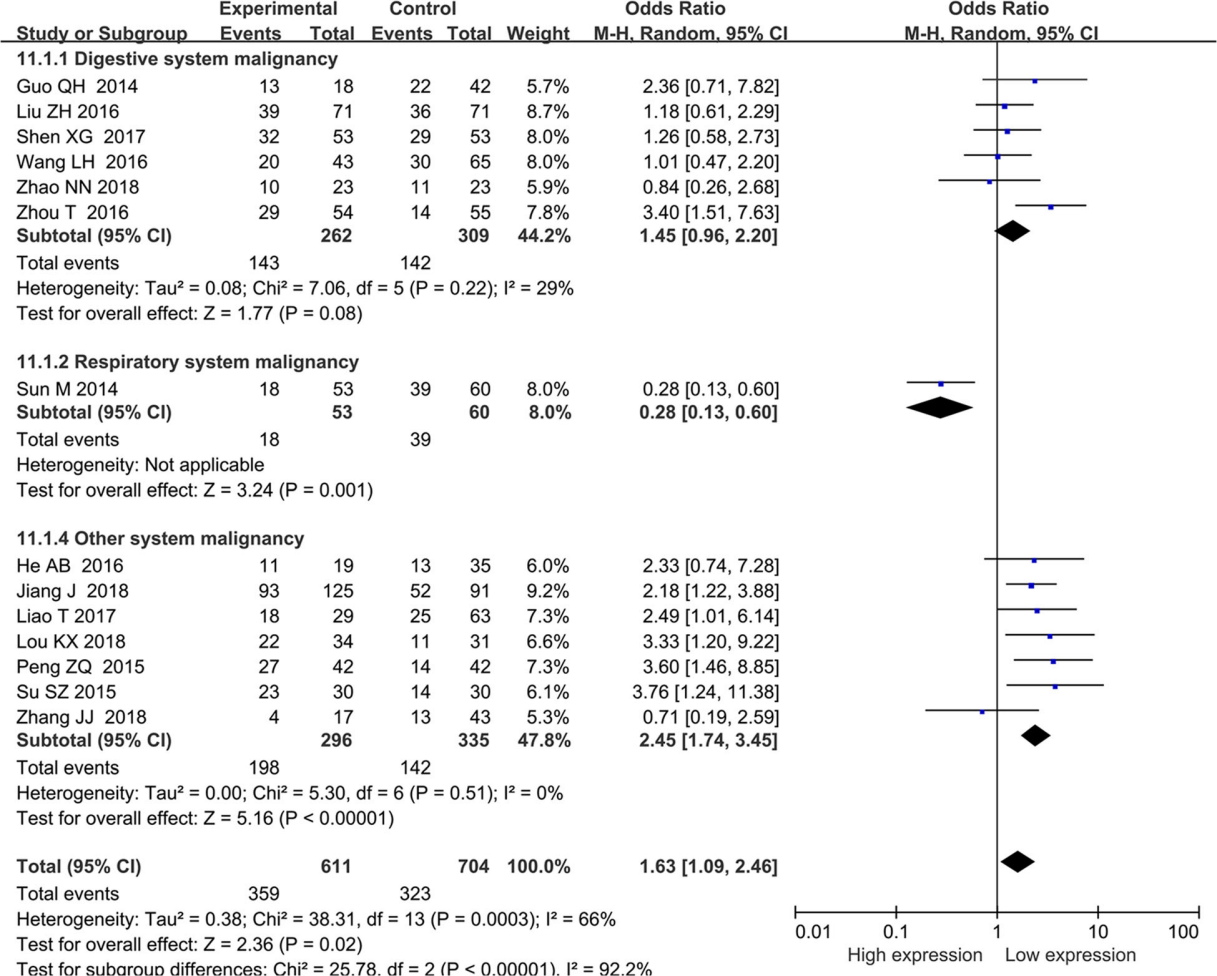
Forest plot of the relationship between BANCR expression and tumor size. Note: BANCR: BRAF-activated noncoding RNA; CI: confidence interval; Random: random-effects model. The random-effects model was adopted. The square size of individual studies represented the weight of the study. Vertical lines represent 95% CI of the pooled estimate. The diamond represents the overall summary estimate, with the 95% CI given by its width

Other prognostic parameters were also assessed, and obvious correlations between increased BANCR expression and advanced lymph node metastasis (OR = 2.03, 95% CI = 1.08–3.83, *P* < 0.05) (Fig. 4), distant metastasis of tumor cells (OR = 3.08, 95% CI: 1.92–4.96, *P* < 0.001) (Fig. 5a), advanced invasion depth (OR = 1.54, 95% CI: 1.06–2.24, *P* = 0.02) (Fig. 5b), worse histological grade (OR = 1.54, 95% CI: 1.00–2.383, *P* = 0.05) (Fig. 5c), larger tumor size (OR = 1.63, 95% CI: 1.09–2.46, *P* = 0.02) (Fig. 6) and more local tumor nodes (multiple / single) (OR = 1.78, 95% CI: 1.12–2.83, *P* = 0.01) were found. However, no associations were found for smoking status (smoker vs. nonsmoker) (OR = 1.01, 95% CI: 0.65–1.56, *P* = 0.98), age (old vs. young) (OR = 0.88, 95% CI: 0.71–1.09, *P* = 0.236) and sex (female vs. male) (OR = 0.91, 95% CI: 0.72–1.16, *P* = 0.469) (Table 4).

**Table 4:** Pool effects of Clinicopathologic characteristics in cancer patients with abnormal BANCR expression

Clinicopathologic characteristics	No. of studies	No. of patients	Odds ratio (95% CI)		*P*	Heterogeneity	
			Fixed	Random		I2(%)	*P*-value
**Age**	15	1469	0.88 (0.71–1.09)	0.88 (0.71–1.09)	0.236	0.0	0.672
**gender**	13	1218	0.91 (0.72–1.16)	0.91 (0.70–1.18)	0.469	9.1	0.355
**TNM (III + IV vs. I + II)**	14	1378	2.27 (1.82–2.84)	2.39 (1.26–4.53)	0.008	83.9	0.000
Digestive system	7	724	3.69 (2.67–5.10)	4.01 (2.45–6.57)	0.0001	49	0.07
Respiratory system	1	113	0.26 (0.11–0.60)	0.26 (0.11–0.60)	0.002	–	–
Female reproductive system	1	30	12.25 (1.27–118.36)	12.25 (1.27–118.36)	0.03	–	–
Other system malignancy	5	511	1.89 (1.30–2.73)	1.34 (0.38–4.79)	0.65	88	0.000
**LNM (present vs. absent)**	12	1226	2.09 (1.65–2.64)	2.03(1.08–3.83)	0.028	82.2	0.000
Digestive system	5	600	3.35 (2.38–4.72)	3.41 (2.32–5.00)	0.00001	15	0.320
Respiratory system	1	113	0.28 (0.13–0.61)	0.28 (0.13–0.61)	0.001	–	–
Female reproductive system	1	30	9.33 (0.96–90.94)	9.33 (0.96–90.94)	0.05	–	–
Other system malignancy	5	483	1.92 (1.30–2.84)	1.30 (0.43–3.94)	0.64	82	0.000
**Tumor size (big vs small)**	14	1325	1.56 (1.25–1.95)	1.63(1.09–2.46)	0.020	66.0	0.000
Digestive system	6	571	1.45 (1.04–2.03)	1.45 (0.96–2.20)	0.080	29.0	0.220
Respiratory system	1	113	0.28 (0.13–0.60)	0.28 (0.13–0.60)	0.001	–	–
Other system malignancy	7	631	2.44 (1.74–3.41)	2.45 (1.74–3.45)	0.000	0.0	0.510
**Histological grade**	10	830	1.47(1.10–1.97)	1.54 (1.00–2.38)	0.050	44.0	0.060
Digestive system	6	646	1.28 (0.92–1.78)	1.25 (0.72–2.17)	0.440	57.0	0.040
Non-digestive system	4	174	2.45 (1.30–4.63)	2.43 (1.28–4.63)	0.007	0.0	0.660
**DM (present vs. absent)**	4	485	3.08 (1.92–4.96)	2.87 (1.58–5.21)	0.001	23.0	0.273
**Invasion depth (T3 + T4/T1 + T2)**	4	534	1.54 (1.06–2.24)	1.37(0.66–2.83)	0.02	69	0.020
**smoking (smoker vs. non-smoker)**	3	330	1.01 (0.65–1.56)	1.01 (0.56–1.82)	0.98	41.0	0.184
**local tumors (multiple/total)**	4	355	1.78 (1.12–2.83)	1.89 (0.95–3.74)	0.07	45	0.140

Note: *BANCR* BRAF-activated noncoding RNA; *LNM* Lymph node metastasis; *Random* Random-effect model; *TNM* TNM stage;

*DM* Distant metastasis; *Fixed* Fixed-effect model

### Publication bias and sensitivity analysis

**Fig. 7:**
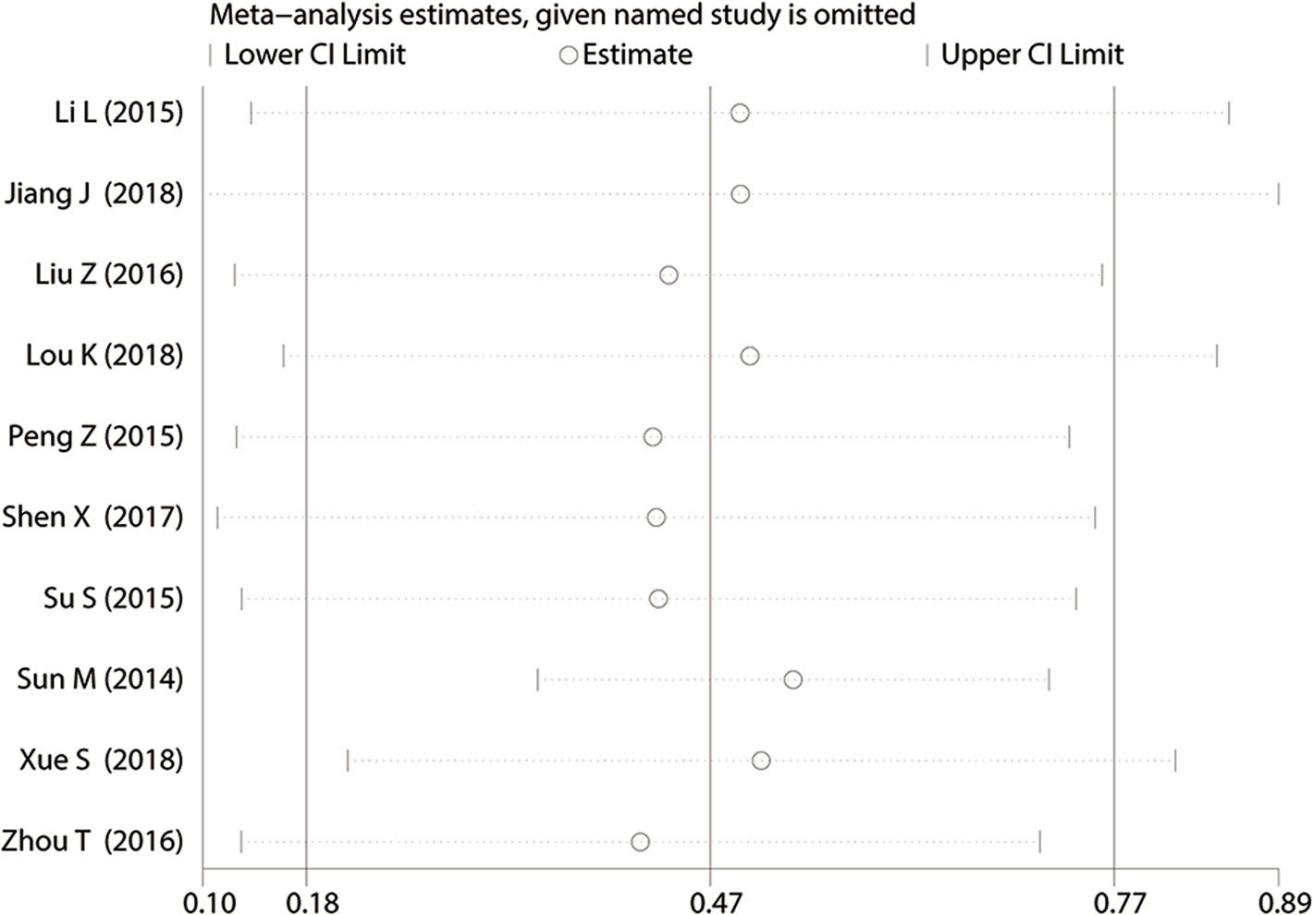
Sensitivity analysis for the association of BANCR expression with overall survival (OS) in various cancers. BANCR: BRAF-activated noncoding RNA; HR: hazard ratio; CI: confidence interval

**Fig. 8:**
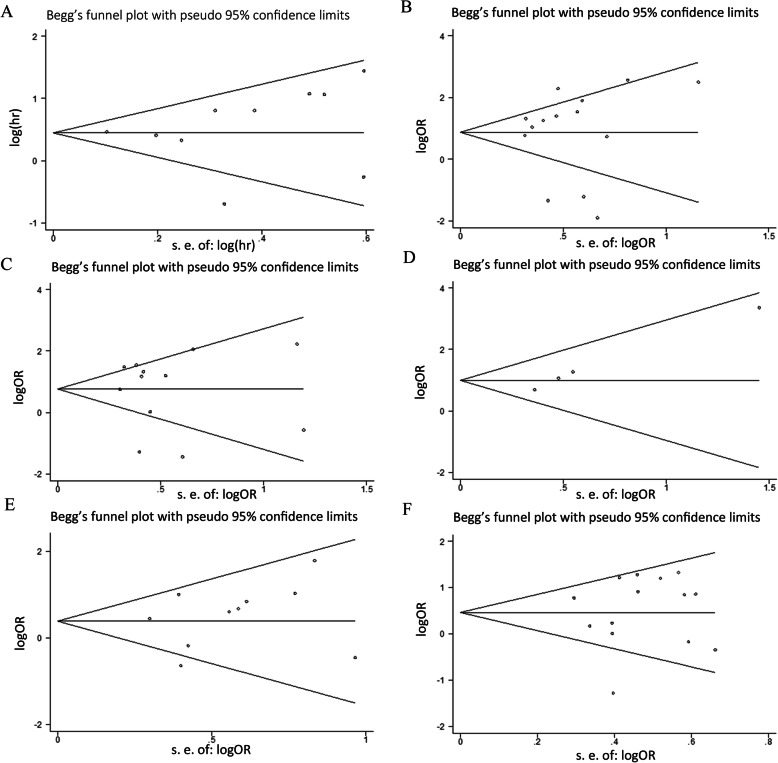
Funnel plot for the correlation between BANCR expression and different prognostic indicators. Note: **a** Overall survival. **b** TNM stage. **c** Lymph node metastasis. **d** Distant metastasis. **e** Depth of invasion. **f** Tumor size. BANCR: BRAF-activated noncoding RNA; OR: odds ratio

Sensitivity analysis was performed to assess the OS outcome stability among the included studies. We found that removing each study successively did not influence the overall results significantly (The overall HR value of the sensitivity analysis is: HR = 0.47, 95% CI: 0.18–0.77. The detail HR value with removing each study successively could be seen in Fig. 7, and no HR value exceeds the confidence interval of the combining result (95% CI: 0.18–0.77)), indicating that the results of each publication were almost consistent with the combined results, in other words, the merged results have high robustness and reliability (Fig. 7). Potential publication bias was estimated by Begg’s test. As shown in Fig. 8, slight publication bias was revealed among the included studies for OS (*Pr* > |*z*| =0.245), TNM stage (*Pr* > |*z*| =0. 477), LNM (*Pr* > |*z*| =0. 493), DM (*Pr* > |*z*| =0. 042), histological grade (*Pr > |z| =* 0.245) and tumor size (*Pr* > |*z*| =0.497). Consequently, there was no significant publication bias in this meta-analysis.

## Discussion

BRAF-activated noncoding RNA (BANCR) was first found in melanoma cells by Flockhart RJ et al. and was reported to be involved in the occurrence and development of diseases, such as coronary artery disease, diabetic retinopathy and cancer [37, 40, 41]. After several years of investigation, an increasing number of studies have reported that BANCR could serve as both an oncogene and tumor suppressor gene in various cancers [15, 19, 39]. In addition, a growing body of literature has reported that aberrant BANCR expression could be detected in breast cancer, gastric cancer, esophageal cancer, hepatocellular carcinoma, endometrial cancer, retinoblastoma and osteosarcoma. High BANCR expression predicts poor survival outcomes, advanced TNM stages, positive lymph node metastasis, poor histological grade and earlier distant metastasis of tumor cells. However, several publications have shown that BANCR could act as a favorable prognostic factor in non-small cell lung cancer and renal carcinoma.

Based on the conflicting conclusions, some researchers tried to explore the potential molecular biological mechanisms of BANCR in the occurrence and development of cancer (Table 5). Flockhart et al. reported that the knockdown of BANCR may significantly downregulate the expression of 86 genes that are closely related to the migration and proliferation of tumor cell [41]. Su et al. detected high BANCR expression in retinoblastoma cells and confirmed that elevated BANCR expression promotes the proliferation, migration and invasion of retinoblastoma cells [38]. Wang et al. found that high BANCR expression could be observed in HCC tissues and that high BANCR may induce the proliferation and invasion of liver cancer cells by inhibiting E-cadherin expression and promoting Vimentin expression. Zhang et al. suggested that downregulated BANCR expression drives aggressiveness in papillary thyroid cancer through the MAPK and PI3K pathways [26]. Lou et al. confirmed that the knockdown of BANCR expression could inhibit the proliferation and induce the apoptosis of breast cancer cells by promoting the epithelial-mesenchymal transition (EMT) process [33]. Additionally, it has been reported that the expression of BANCR is increased in colorectal cancer (CRC) and that BANCR could strengthen the migration and proliferation abilities of CRC by inducing epithelial-mesenchymal transition (EMT) via the activation of the MEK/ERK signaling pathway [34, 42]. Conversely, Liao et al. discovered that in papillary thyroid cancer (PTC) patients, the expression of BANCR was downregulated, which partially suppressed the proliferation, migration and invasion of PTC cells via the ERK/MAPK signaling pathway [24]. Likewise, Sun et al. observed a decreased expression of BANCR in NSCLC cells, and low BANCR expression may drive NSCLC cell invasion and metastasis by affecting EMT. In summary, the expression level and role of BANCR varies from cancer to cancer, possibly due to the differences between tumors. A comprehensive analysis is therefore needed to accurately assess the prognostic value of BANCR in cancer.

**Table 5:** Transition of cell phenotype and related molecular mechanisms with abnormal BANCR expression in various cancers

Cancer type	Expression	Micro-RNAs	Targets	Functions	References
non-small cell lung cancer	down-regulation	–	MMP2; MMP9; N-cadherin; E-cadherin	epithelial-mesenchymal transition (EMT)	[19]
hepatocellular carcinoma	up-regulation	–	Vimentin; E-Cadherin	migration, invasion	[17]
	up-regulation	–	Bcl-2; Bax; MEK; ERK; JNK; P38;	cell invasion, proliferation and migration and apoptosis	[14]
	up-regulation	–		cell proliferation and migration	[15]
	up-regulation	–	–	cell growth, migration and invasion	[16]
osteosarcoma	up-regulation	–	ZEB1	apoptosis	[11]
papillary thyroid cancer	down-regulation	–	AKT; MEK; ERK; JNK; P38;	proliferation, migration and invasiveness	[24]
	down-regulation	–	MAPK; PI3K-AKT	cell growth, cycle and apoptosis	[25, 26]
	up-regulation	–	Raf; MEK; ERK;	cell autophagy	[27]
colorectal cancer	up-regulation	–	Vimentin; E-Cadherin; MEK; ERK;	epithelial-mesenchymal transition (EMT)	[34]
	up-regulation	miR-203	CSE1L	proliferation and invasion; cell sensitivity to adriamycin (ADR)	[42]
bladder cancer	down-regulation	–	–	apoptosis and migration	[30]
Malignant Melanoma	up-regulation		AKT; MEK; ERK; JNK; P38;	cell proliferation and migration	[31]
breast cancer	up-regulation	–	Bcl-2; Bax; PARP; Cleaved-caspase3	cell proliferation and invasion	[33]
	up-regulation	–	Vimentin; E-Cadherin; MMP2; MMP9; MMP14	cell migration and invasion	[32]
clear cell renal cell carcinoma	up-regulation	–	caspase3; caspase9; CDK4; CDK6	cell growth, cycle and apoptosis	[18]

**Note:** BRAF-activated noncoding RNA; MMP2, The matrix metalloproteinases 2; MMP9, The matrix metalloproteinases 9; MMP14: The matrix metalloproteinases 14; PARP: poly ADP-ribose polymerase; EMT, Epithelial-Mesenchymal Transition; ZEB1, zinc finger E-box binding homeobox 1; MAPK: Mitogen-activated protein kinase; ERK: extracellular signal-regulated kinase; JNK: Jun N-terminal kinases; CDK4: cyclin-dependent kinase 4; CDK6: cyclin-dependent kinase 6; NA, Not Available

Considering the varied conclusions mentioned above, 20 studies with 1997 patients and 12 types of cancers were finally enrolled in this meta-analysis to explore the relationship between BANCR expression level and the prognosis of cancer patients. The pooled HR showed a marked association between high BANCR expression and worse OS. Considering the underlying heterogeneity and different expression levels of BANCR, a subgroup analysis according to cancer type, HR estimation method, the expression levels of BANCR, NOS scores and sample size was conducted to investigate the sources of heterogeneity, and obvious associations were found for the digestive system (HR = 1.87, 95% CI, 1.40–2.50, *P* < 0.0001), HRs extracted directly from articles (HR = 1.69, 95% CI, 1.44–1.99, *P* < 0.0001), HRs from multivariate analysis (HR = 1.79, 95% CI, 1.47–2.18, *P* < 0.00001), high BANCR expression group (HR = 1.72, 95% CI, 1.48–1.98; *P* < 0.00001), studies with fewer than 100 patients (HR = 1.71, 95% CI, 1.01–2.90, *P* = 0.01) and studies with more than 100 patients (HR = 1.57, 95% CI, 1.07–2.31, *P* = 0.01). On the other hand, through subgroup analysis, we can observe that the heterogeneities of some subgroups reduced significantly heterogeneity (Table 3), such as digestive system (*I*^2^ = 17%), other systems (*I*^2^ = 15%), multivariate analysis (*I*^2^ = 11%), direct HR extraction (*I*^2^ = 0%), and less than 100 subjects (*I*^2^ = 36%). Low heterogeneity suggests reliability, stability and persuasive of results. The unfavorable survival prognosis related to BANCR in cancers was also confirmed for RFS (HR = 1.88, 95% CI: 1.09–3.25). However, no associations were found between BANCR expression and OS for non-digestive system cancers (HR = 1.35, 95% CI, 0.86–2.13; *P* = 0.20), HRs from univariate analysis (HR = 0.84, 95% CI, 0.41–1.75, *P* = 0.78) or HRs extracted indirectly from articles (HR = 1.15, 95% CI, 0.52–2.56, *P* = 0.69). In addition, high BANCR expression was observed to be related to advanced clinical stage (OR = 2.39, 95% CI: 1.26–4.53, *P* = 0.008), lymph node metastasis (OR = 2.03, 95% CI: 1.08–3.83, *P* = 0.03), distant metastasis (OR = 3.08, 95% CI: 1.92–4.96, *P* < 0.00001), more local tumor nodes (OR: 1.78, 95% CI: 1.12–2.83, *P* = 0.01) (**Figure** **S1**), and larger tumor sizes (OR: 1.63, 95% CI: 1.09–2.46, *P* = 0.02) but was not related to smoking status (OR: 1.01, 95% CI: 0.65–1.56, *P* = 0.98) (**Figure** **S2**), age (OR: 0.88, 95% CI: 0.71–1.09, *P* = 0.236) (**Figure** **S3**) or sex (OR: 0.91, 95% CI: 0.72–1.16, *P* = 0.469) (**Figure** **S4**). In summary, despite serving as both an oncogene and a tumor suppressor gene in different cancers, the pooled results still support the conclusions of most primary studies that have shown that high BANCR expression indicates worse cancer prognosis. The results of the sensitivity analysis for OS showed that the overall results were not significantly affected by the arbitrary deletion of a certain study, which supported the stability of the results. In addition, slight publication bias was observed in the included studies. Therefore, the expression level of BANCR could be used to evaluate the prognosis of tumor patients in most cancers.

Although the relationship between BANCR expression and clinical prognosis has been assessed by Hu et al. and Fan et al. 　[43, 44], there are several differences between these previous investigations and our research. First, the pooled results revealed the significant association between high BANCR expression and worse OS and RFS, advanced TNM stage and a high risk of lymph node metastasis, which failed to be concluded by a previous meta-analysis. Second, larger sample sizes and more cancer types were included in this meta-analysis. Third, comprehensive subgroup analysis was performed, and the correlations between BANCR and tumor size, histological grade, invasion depth, smoking status, number of local tumors, age and sex were first explored in this study, which were not investigated in the previous meta-analysis. Finally, the detailed molecular biological mechanisms of BANCR in various cancers were discussed and summarized. Nevertheless, there are some limitations in this meta-analysis: (a) most of the patients included in this study came from China, which may limit the generalizability of the results; (b) the sample size included was not large enough, which may affect the reliability of the results; (c) only 11 types of cancers were included to investigate the association between BANCR and cancer prognosis; thus, the conclusions of this study could not represent all cancers; (d) some HR values were extracted from survival curves, which may partly lead to extraction bias.

## Conclusion

In general, the high expression of BANCR is significantly associated with shorter OS and poor clinical prognosis, and BANCR may be treated as a biomarker and therapeutic target for cancer. High quality, larger sample size and multicenter studies are needed to further confirm the reliability of this conclusion.

## Supplementary information

**Additional file 1: Figure S1.** Forest plot of the relationship between BANCR expression and the number of local tumors (multiple/single). Note: BRAF-activated noncoding RNA; OR: odds ratio; CI: confidence interval; Random: random-effects model. The random-effects model was adopted. The square size of individual studies represented the weight of the study. Vertical lines represent 95% CI of the pooled estimate. The diamond represents the overall summary estimate, with the 95% CI given by its width

**Additional file 2: Figure S2.** Forest plot of the relationship between BANCR expression and smoking status (smoker vs. nonsmoker). Note: BANCR: BRAF-activated noncoding RNA; OR: odds ratio; CI: confidence interval; Fixed: fixed-effects model. The fixed-effects model was adopted. The square size of individual studies represented the weight of the study. Vertical lines represent 95% CI of the pooled estimate. The diamond represents the overall summary estimate, with the 95% CI given by its width

**Additional file 3: Figure S3.** Forest plot of the relationship between BANCR expression and age (older vs. young). Note: BRAF-activated noncoding RNA; OR: odds ratio; CI: confidence interval; Fixed: fixed-effects model. The fixed-effects model was adopted. The square size of individual studies represented the weight of the study. Vertical lines represent 95% CI of the pooled estimate. The diamond represents the overall summary estimate, with the 95% CI given by its width

**Additional file 4: Figure S4.** Forest plot of the relationship between BANCR expression and sex (female vs. male). Note: BRAF-activated noncoding RNA; OR: odds ratio; CI: confidence interval; Fixed: fixed-effects model. The fixed-effects model was adopted. The square size of individual studies represented the weight of the study. Vertical lines represent 95% CI of the pooled estimate. The diamond represents the overall summary estimate, with the 95% CI given by its width

## Data Availability

All data generated or analysed during this study are included in this published article.

## Abbreviations

BANCR: BRAF-activated noncoding RNA
LncRNAs: Long non-coding RNAs
NSCLC: Non-small cell lung cancer
HCC: Hepatocellular carcinoma
CRC: Colorectal cancer
BL: Bladder cancer
BC: Breast cancer
ccRCC: Clear cell renal cell carcinoma
GC: Gastric cancer
LNM: Lymph node metastasis
DM: Distant metastasis
HTS: High tumor stage (III, IV)
NA: Not available
qRT-PCR: Quantitative reverse transcription-polymerase chain reaction
ESCC: Esophageal cancer
EC: Endometrial cancer
SC: Survival curve
directly: The HR was extracted directly from the article
PTC: Thyroid carcinoma
OS: Overall survival
DFS: Disease-free survival
RFS: Recurrence-free survival
OR: Odds ratio
HR: Hazard ratio
NOS: Newcastle-Ottawa scale
MMP2: Matrix metalloproteinase 2.
MMP9: Matrix metalloproteinase 9.
EMT: Epithelial-mesenchymal transition.
ZEB1: Zinc finger E-box binding homeobox 1.
MAPK: Mitogen-activated protein kinase
ERK: Extracellular signal-regulated kinase
JNK: Jun N-terminal kinase
Random: Random-effects model
TNM: TNM stage
Fixed: Fixed-effects model
