# Supplementary figures and images for: High BANCR expression is associated with worse prognosis in human malignant carcinomas: an updated systematic review and meta-analysis
Source: BMC Cancer. 2020 Sep 9;20:870. doi: 10.1186/s12885-020-07177-6 (PMC7488167; doi:10.1186/s12885-020-07177-6)

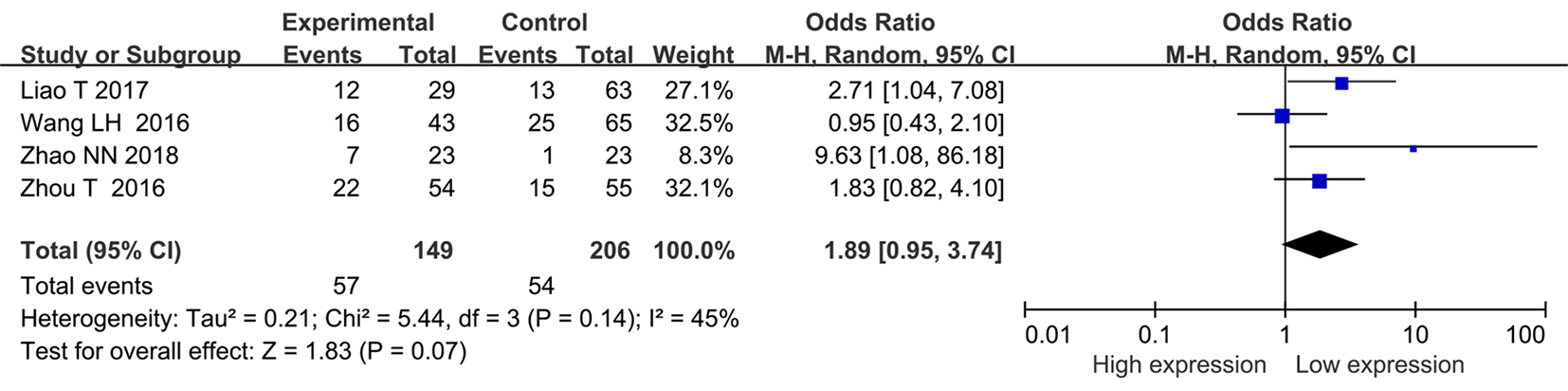

Supplement: Supplementary file 1 — Additional file 1: Figure S1. Forest plot of the relationship between BANCR expression and the number of local tumors (multiple/single). Note: BRAF-activated noncoding RNA; OR: odds ratio; CI: confidence interval; Random: random-effects model. The random-effects model was adopted. The square size of individual studies represented the weight of the study. Vertical lines represent 95% CI of the pooled estimate. The diamond represents the overall summary estimate, with the 95% CI given by its width [file 12885_2020_7177_MOESM1_ESM.tif]

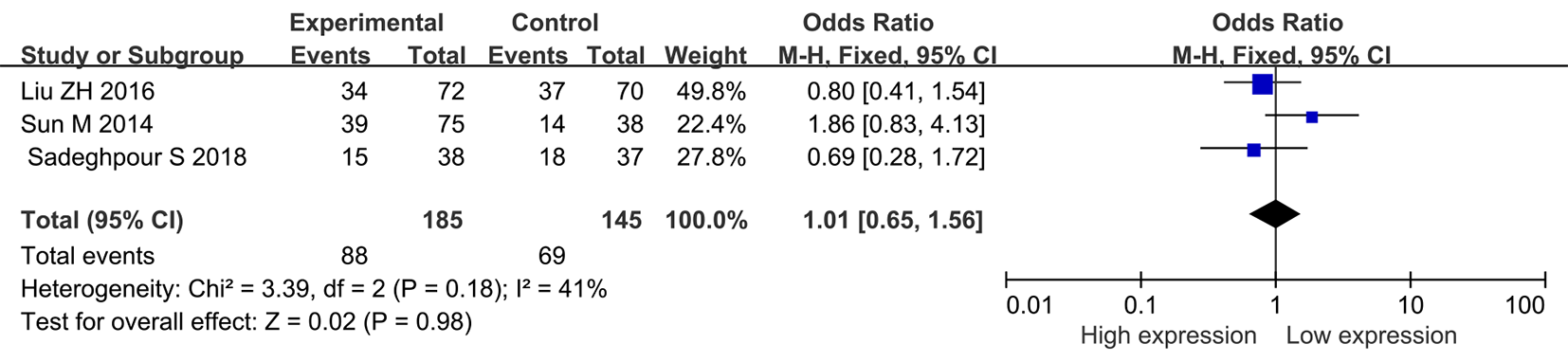

Supplement: Supplementary file 2 — Additional file 2: Figure S2. Forest plot of the relationship between BANCR expression and smoking status (smoker vs. nonsmoker). Note: BANCR: BRAF-activated noncoding RNA; OR: odds ratio; CI: confidence interval; Fixed: fixed-effects model. The fixed-effects model was adopted. The square size of individual studies represented the weight of the study. Vertical lines represent 95% CI of the pooled estimate. The diamond represents the overall summary estimate, with the 95% CI given by its width [file 12885_2020_7177_MOESM2_ESM.tif]

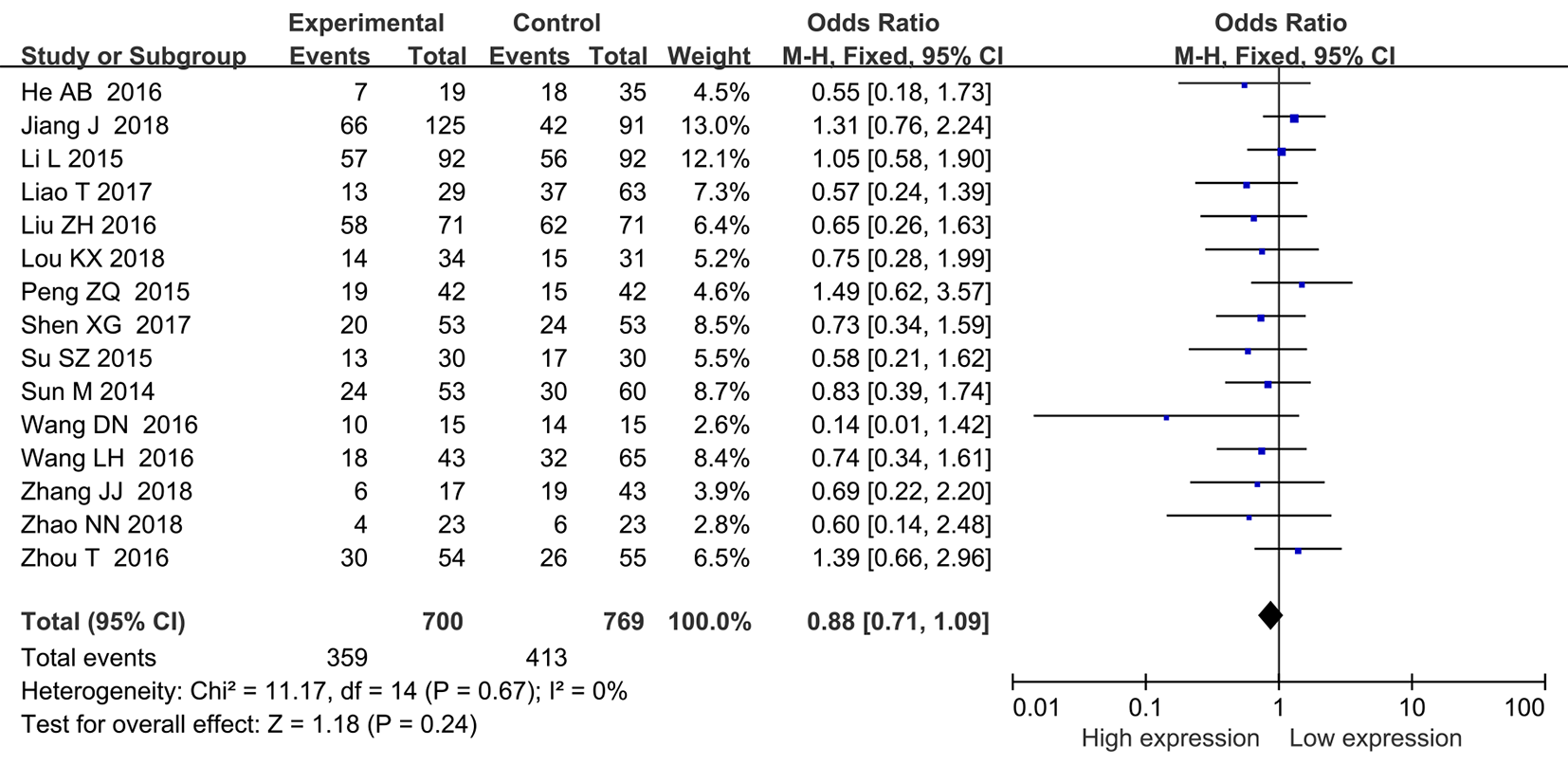

Supplement: Supplementary file 3 — Additional file 3: Figure S3. Forest plot of the relationship between BANCR expression and age (older vs. young). Note: BRAF-activated noncoding RNA; OR: odds ratio; CI: confidence interval; Fixed: fixed-effects model. The fixed-effects model was adopted. The square size of individual studies represented the weight of the study. Vertical lines represent 95% CI of the pooled estimate. The diamond represents the overall summary estimate, with the 95% CI given by its width [file 12885_2020_7177_MOESM3_ESM.tif]

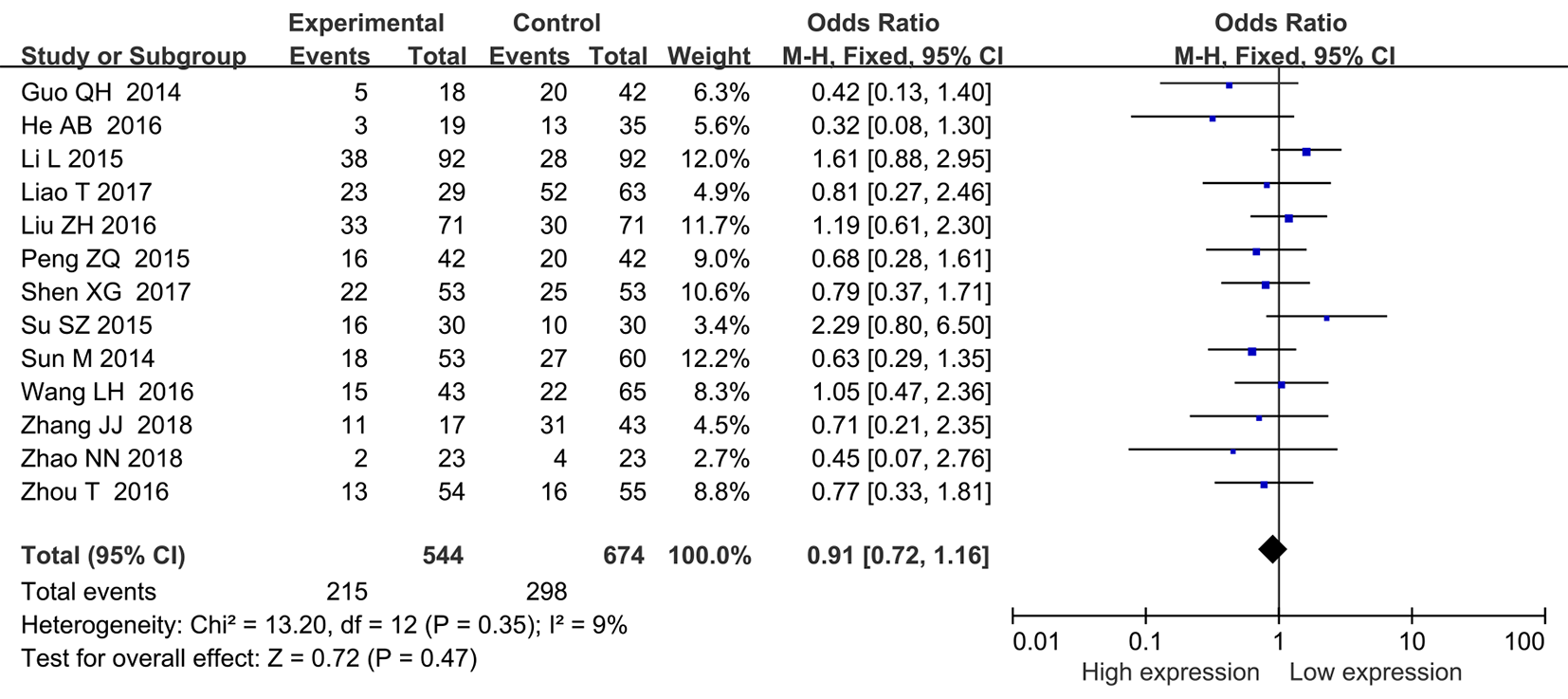

Supplement: Supplementary file 4 — Additional file 4: Figure S4. Forest plot of the relationship between BANCR expression and sex (female vs. male). Note: BRAF-activated noncoding RNA; OR: odds ratio; CI: confidence interval; Fixed: fixed-effects model. The fixed-effects model was adopted. The square size of individual studies represented the weight of the study. Vertical lines represent 95% CI of the pooled estimate. The diamond represents the overall summary estimate, with the 95% CI given by its width [file 12885_2020_7177_MOESM4_ESM.tif]
